# Spinal fMRI of Interoceptive Attention/Awareness in Experts and Novices

**DOI:** 10.1155/2014/679509

**Published:** 2014-06-17

**Authors:** Keyvan Kashkouli Nejad, Motoaki Sugiura, Benjamin Thyreau, Takayuki Nozawa, Yuka Kotozaki, Yoshihito Furusawa, Kozo Nishino, Toshohiro Nukiwa, Ryuta Kawashima

**Affiliations:** ^1^Department of Functional Brain Imaging, Institute of Development, Aging and Cancer, Tohoku University, Seiryo-machi 4-1, Aoba-ku, Sendai 980-8575, Japan; ^2^International Research Institute of Disaster Science, Tohoku University, Sendai 980-8575, Japan; ^3^Tohoku Medical Megabank Organization, Tohoku University, Sendai 980-8575, Japan; ^4^Department of Physical Medicine and Rehabilitation, Graduate School of Medicine, Tohoku University, Sendai 980-8575, Japan; ^5^Institute of Nishino Breathing Method, Tokyo 150-0046, Japan; ^6^Department of Respiratory Medicine, Tohoku University Graduate School of Medicine, Sendai 980-8574, Japan; ^7^South Miyagi Medical Center, Miyagi, Shibata 989-1253, Japan

## Abstract

Many disciplines/traditions that promote interoceptive (inner sensation of body parts) attention/awareness (IAA) train practitioners to both attend to and be aware of interoceptive sensory experiences in body parts. The effect of such practices has been investigated in previous imaging studies but limited to cerebral neural activity. Here, for the first time, we studied the impact of these practices on the spinal neural activity of experts and novices. We also attempted to clarify the effect of constant and deep breathing, a paradigm utilized in concentration practices to avoid mind wandering, on IAA-related spinal neural activity. Subjects performed IAA tasks with and without a deep and constant breathing pattern in two sessions. Results showed that neural activity in the spinal segment innervating the attended-to body area increased in experts (*P* = 0.04) when they performed IAA and that this increase was significantly larger for experts versus novices in each of the sessions (*P* = 0.024). The significant effects of IAA and expertise on spinal neural activity are consistent with and elaborate on previous reports showing similar effects on cerebral neural activity. As the spinal cord directly innervates body parts, the results might indicate that IAA has an instantaneous (possibly beneficial) effect on the physical body after extended training.

## 1. Introduction

Many disciplines/traditions (e.g., Chinese Qi, Japanese Kokyuho, Iranian Movazeneh, and Indian Yoga) train practitioners to attend to and be aware of the visceral sensory experiences of body parts. For example, a practitioner may be instructed to feel or even activate the flow of energy in discrete body parts, such as the tip of the nose, throat, and heart, or may be asked to scan the whole body by attending to each part slowly and continuously. Such practices are referred to as interoceptive attention/awareness (hereafter, IAA) [1–3] or mindfulness (e.g., mindfulness of breathing [4, 5]). There are different reasons for the use of such practices, but one simple motivation is that they are beneficial for mental and physical health. Various studies have investigated the neural correlates or impact of such practices on the brain [4, 6–9], and results show structural changes in cortical areas (e.g., increased regional gray matter concentration in the right anterior insula, which is involved in interoceptive awareness) as well as functional alterations (e.g., decreased default-mode-network activity) among those who are expert in such practices. Vestergaard-Poulsen et al. [10] examined the impact of such training in the brainstem and showed that long-term concentration practice is associated with gray matter density in the lower brain stem.

We investigated the neural impact of IAA practices on the spinal cord by comparing the spinal cord neural activity of expert with novice practitioners using MRI. Such investigation may provide further evidence related to the belief that these practices are beneficial for physical health [11–14] as the spinal cord is directly connected to the body in that it innervates each part via various spinal nerve segments (e.g., the cervical cord innervates the hands and arms, and the lumbar segment innervates the feet and legs). The spinal cord was once considered as a mere relay that executes pregenerated plans originating in supraspinal structures or delivers sensory afferent signals from the periphery, but this view has been challenged in various studies and thus no more holds to be true. For example, Prut and Fetz [15] demonstrated spinal cord activation during motion planning and argued that motor cortical areas may interact continuously with spinal networks, possibly to modulate the transmission/effect of efferent (afferent) information from supraspinal levels on the periphery (and vice versa). Similarly, attention to body parts may involve both the manipulation of neural signals inside the brain as well as the modulation of interactions between the spinal cord segment innervating the target body part and the relevant supraspinal regions.

We believe that the consistent ability to modulate such neural interactions through IAA requires considerable expertise. In fact, most disciplines believe that long-term training and devotion are needed to achieve the ability to practice optimally [16, 17]. Such a requirement for expertise can also be inferred from previous studies, which indicated that novices exhibit more mind wandering than do expert practitioners when performing attention/meditation tasks [7]. Hence, we expected experts would demonstrate more spinal cord neural activity than would novices when attending to body parts. We also studied the effect of a constant breathing paradigm, a strategy used to enhance the outcome of the practices, on the spinal neural activity related to the IAA task. Deep and constant breathing rids the brain of distorted thoughts and prepares it for concentration [18]. This concept is supported by previous neuroimaging studies on mindfulness breathing [5]. Therefore, we expected that application of the controlled-breathing paradigm would enhance the effect of IAA on spinal neural activity. On the other hand, similar to IAA, this strategy requires training to be performed properly and, in some disciplines, students perform breathing exercises for a few weeks or months before they progress to other attention/awareness exercises. Therefore, we expected that the interaction between expertise and breathing method would have a positive effect on spinal activity due to experts' additional practice of controlled-breathing patterns in conjunction with IAA.

For this experiment, we chose a body part with a relatively high potential to be better attended to by experts compared with novices: the pubic/lower abdominal region, which is more highly emphasized in East Asian practices than are other parts of the body. Although the motivation for this emphasis is based more on tradition than established medical knowledge, experts recruited in this study have been extensively trained to concentrate on this body part. As the nerves innervating this region arise primarily from L1-2 lumbar segments [19], the region of interest in this study included those spinal cord segments.

To the best of our knowledge, this is the first time that the impact of IAA training on spinal cord neural activity has been investigated using MRI. Previous studies provide us with valuable evidence regarding feasibility of the spinal fMRI in different parts of spinal cord (cervical or even lumbar areas [20]) and its applicability for group studies [21]. Additionally, some studies examined the effect of attention on motion- and sensory-related neural activation; for example, [22] investigated the effect of attention versus distraction to the thermal stimulation of the body, or Smith and Kornelsen [23] compared the effect of different emotional stimuli on spinal neural activity during motion tasks. However, all previous studies investigated spinal neural activity in the presence of sensory stimuli [21, 24] or during the performance of motion tasks [25, 26].

## 2. Materials and Methods

There are fewer spinal fMRI experiments than there are studies of the brain due primarily to the technical challenges involved in spinal fMRI. The main problem in this regard involves the huge signal dropout in conventional T2* imaging due to differences in the magnetic susceptibility of the bones, cartilage and tissues, and air-filled lungs [27] near the spinal cord. Therefore, we used T2 imaging, which does not suffer from signal dropout and has been tested in previous studies [28]. Other problems related to spinal fMRI include the long length and small cross-section of the spinal cord [27] and the lack of a standardized coordinate system [29] and automated image-registration toolboxes. In this section, we will describe how we handle these issues.

### 2.1. Participants

The experimental protocol was approved by the Ethics Committee of Tohoku University Graduate School of Medicine. Written informed consent was obtained from each subject. Expert (*E*) subjects were recruited from Institute of Nishino Breathing Method [30]. The expert group consisted of nine male participants with a mean age of 56.9 years (standard deviation, SD: 11.1 years) and an average of 16 (SD: 8.2) years of IAA experience. This group included subjects who (1) had more than 3 years of experience, (2) had been practicing regularly during the last 6 months, and (3) were either professional trainers or considered to be experts by professional trainers. Novices (*N*) were recruited by advertisements in a local magazine. The novice group was age- and sex-matched to the expert group and included nine males (for the novice subjects who were used in the analyses, please read below) with a mean age of 55.1 (SD: 7.9) years and no experience with concentration, meditation, or similar disciplines. All subjects were Japanese, and no subject had any history of neurological or psychiatric illness or any auditory problems.

### 2.2. Experimental Overview

The experiment consisted of blocks involving attention to a body part (A) and rest (R). Although all parts of the body are attended to during training, there is a strong emphasis on the pubic/lower abdominal region (it is sometimes considered the reference point in their practices), which is attended to several times in each practice session. Therefore, the pubic/lower abdominal region was selected for attention task, and subjects were told to relax their body and attend to the area about 10 cm below their belly button. Each condition consisted of 24 second blocks, and each block started with a 0.5-s single-word cue that specified the beginning and type of each condition. The length of blocks was kept short to avoid subjects moving or getting tired throughout the scanning. The A and R blocks were alternated, always beginning with an R block.

The experiment was conducted during two sessions. In the normal-breathing (NB) session, subjects were asked to disregard their breathing pattern and simply repeat attention and rest blocks. In the controlled-breathing (CB) session, subjects were asked to match their breathing with the breathing sound that was played while they performed the tasks discussed above. Subjects were instructed to follow the same breathing pattern even during rest. The breathing sound was a recorded breathing cycle with 4 seconds of inhale and 8 seconds of exhale and this sound was amplified and repeatedly played using the computer looping (subjects practiced the task with the breathing before the session; please see below). The breathing sound was adjusted such that all subjects reported that they could hear the breathing cycle and distinguish inhale and exhale. The order of sessions was counterbalanced across subjects. As we were advised by some IAA experts that it would be easier for subjects to attend to their body parts with their eyes closed, all cues and breathing sounds were delivered through headphones, and subjects were asked to close their eyes during the experiment. Subjects practiced each session for about 5 min before the experiment started until all verbally agreed that they understood the tasks. During the attention blocks, subjects were instructed to focus on the task if they found themselves attending to thoughts about topics other than the task.

### 2.3. Behavioral Data

Before each session, subjects were asked to rate their level of sleepiness on a scale from 0 to 10. After each session, subjects left the MRI scanner and were asked to rate their attention to the body part, their sleepiness during the experiment, their degree of mind wandering under each condition (i.e., A and R), and their ability to switch their mental state from attention to rest and vice versa on an 11-point scale (0 to 10). During the CB session, subjects were also asked how well they could match their breathing with the audio sounds.

### 2.4. MRI Data Acquisition

Subjects were scanned on a 3.0 Tesla Philips Achieva MRI scanner. We applied T2-weighted imaging for this study [28]. Using a phased-array spine receiver coil, spinal cord fMRI data were acquired with a single-shot turbo spin-echo (TSE) sequence. Five contiguous sagittal slices with a thickness of 3 mm covering the spinal cord at the level of vertebrae Th9 to L1 were used. Sequence parameters were TR 4310 ms; TE 70 ms (within the range of bold-optimal TE at 3T [28]); flip angle 90 degrees; field of view 200 mm × 100 mm; matrix size 128 × 64. Spatial saturation pulses anterior and posterior to the cord were used in combination with fold-over suppression (NSA = 2, i.e., oversampling twice the size of FOV and discarding the area out of FOV in reconstruction) to avoid aliasing and to reduce artifacts due to breathing motion with a 90 mm slab. Also, to avoid the effect of CSF flow, flow compensation in the readout direction was used. Each session consisted of 116 dynamic volumes, totaling to 232 volumes per subject.

### 2.5. Analyses

We first eliminated subjects who surpassed the sleepiness threshold (subjective sleepiness rating of >7) or who reported low rate of matching their breathing with the audio sounds and/or low rate of switching their attention (<5). Data from one expert during one session were eliminated due to sleepiness in that session. Consequently, the data for the same session of the corresponding age-/sex-matched control subject were removed as well. The novice group previously mentioned only included those who surpassed the above thresholds.

**Figure 1:**
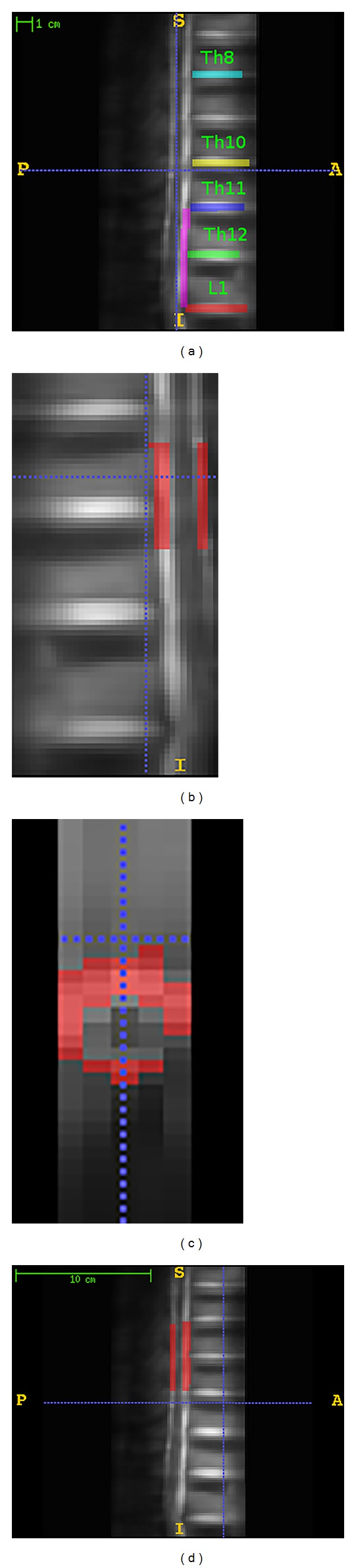
(a) Labeling of the mean T2 images of a representative individual subject in one session. Different colors (intensities) were used for different labels, and each color shows one label. Th denotes the thoracic and L denotes the lumbar vertebrae. (b) Illustration of the ROI, sagittal view. (c) Illustration of the ROI, axial view. (d) Illustration of the secondary mask.

For each session, motion correction of the functional images was first performed using the realignment procedure in the SPM8 toolbox (Wellcome Department of Cognitive Neuroscience, London), which estimates a set of six rigid-body transformation parameters using the first image of each session as the reference image for each subject. The scans were smoothed using a Gaussian kernel set at 8 mm full width at half-maximum. For each subject, a GLM design matrix was constructed, modeling the A condition as a boxcar function convolved with a standard hemodynamic response function (HRF), and the six motion parameters were also included as regressors. Images were normalized into a common space using the Advanced Normalization Tools (ANTs, http://www.picsl.upenn.edu/ANTS/) as follows. First, cushions below the L1, Th12, Th11, Th10, and Th8 vertebrae were manually labeled on the mean functional image for each subject using ITKSNAP (http://www.itksnap.org/pmwiki/pmwiki.php). A sixth label was drawn along the spinal cord adjacent to the vertebrae from the lower part of Th11 to the lower part of L1 (Figure 1(a)). The mean images of all subjects were then warped to a template, which was the mean image of an arbitrary reference subject. This was performed by a series of label-guided affine and nonlinear transformations using the ANTs toolbox (http://www.picsl.upenn.edu/ANTS/) as follows. First, we built two smoothed versions of the label images, one smoothed with a 10 mm kernel and one with a 1 mm kernel. The affine transformation was initialized with transforming the largely smoothed label image of the subject to the smoothed image of the template subject; then this was fed into another layer of affine using the slightly smoothed images, and this was in turn fed into a final affine transformation of the nonsmoothed label images. Finally, the affine transformation was followed by nonlinear transformations converting the mean spinal image of the subject to the mean image of the template subject guided by the labels.

For each subject, contrast images (i.e., A-R map) for each session were normalized by applying the warping parameters used for the mean images.

For the ROI analyses, a mask along the predicted ROI covering the L1-L2 segment was delineated from a template image (nerves innervating the pubic/lower abdominal region mainly arise from L1-2 lumbar segments [19]). This ROI covered the spinal cord starting from the upper third of the T11 vertebrae to below the T11-T12 intervertebral cushion which correspond to L1-L2 spinal segments [31, 32] (the spinal segments and vertebrae are not adjacent). As it can be seen in Figures 1(b) and 1(c) the ROI excluded the spinal canal (where the CSF flows) and also the surface of the spinal cord adjacent to the spinal canal. Also to test if the tasks induce neural activity outside the predicted region of interest, a secondary mask (also excluding the spinal canal) was built starting from below the T8-T9 intervertebral cushion extended until above the T10-T11 intervertebral cushion (Figure 1(d)).

For the second level analysis, we used the normalized contrast images. We first performed used MARSBAR [33] to compute the average signal change across the masks (for both the ROI and the secondary mask) and tested for contrasts of group-wide activation in experts, higher activation in experts compared to novices, and positive interaction of breathing pattern and between sessions across the masks. We used nonparametric Wilcox test and reported results with *P* < 0.05 as significant. We also showed the activation patterns for each of the above mentioned contrasts, where voxels were assessed with *P* < 0.05 (uncorrected), and since there were no significant clusters, cluster extent threshold was set as >30 voxels.

## 3. Results

**Figure 2:**
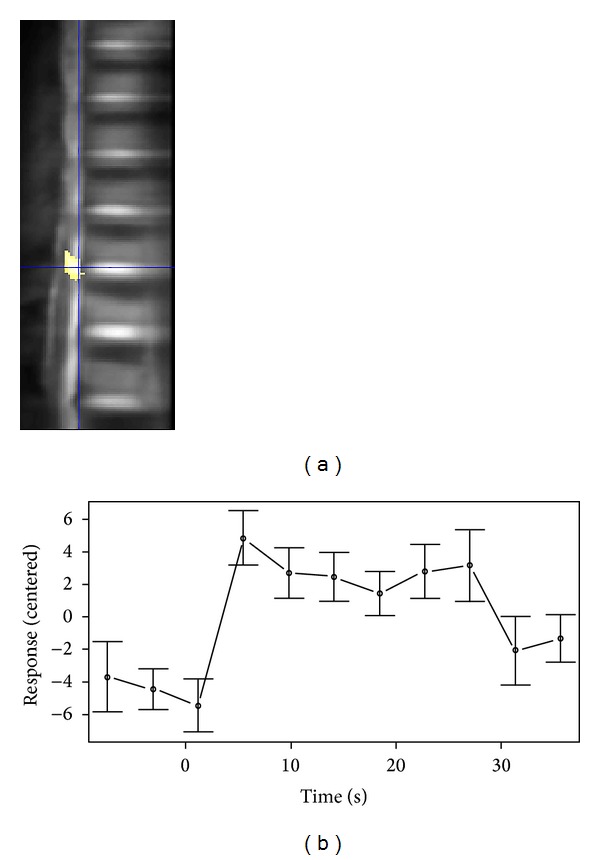
(a) Activation map across the regions of interest for experts; crosshair shows the peak activation voxel. For visualization the spinal canal is not excluded for this map. (b) Per-task response averaged across ten repetitions for one subject. Data was centered; error bars indicate SEM.

Experts showed spinal cord activation in the ROI (*P* = 0.039). As shown in Figure 2(a), the peak of this activation is outside the spinal canal where CSF flows (Figure 2(a) and Table 1).  Figure 2(b) illustrates the per-task response averaged across trials of the task for one subject. As it can be seen there is an increase of signal starting from the onset of the task.

**Figure 3:**
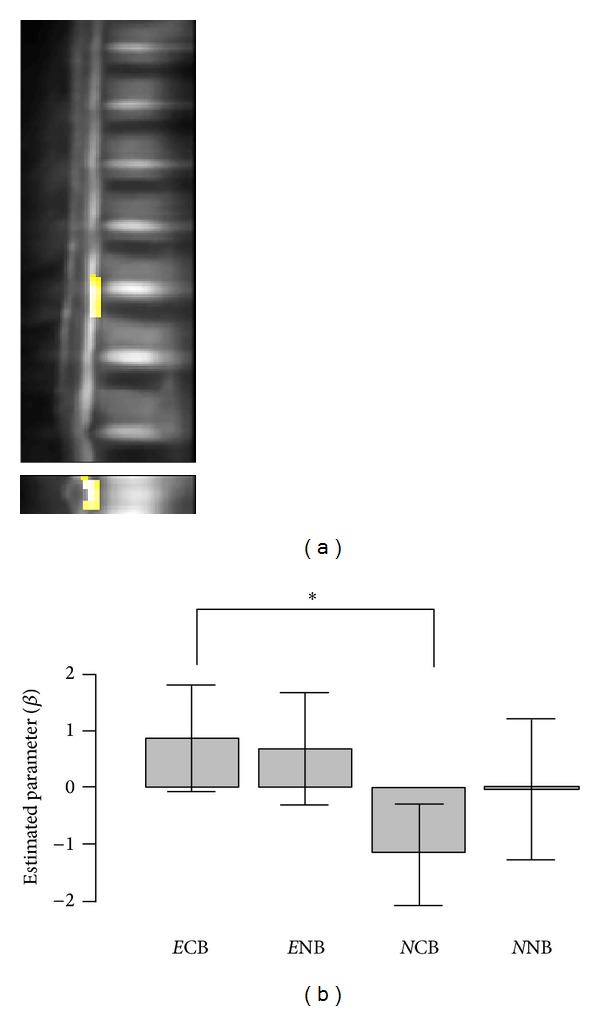
(a) Activation map across the regions of interest for E-N, sagittal and axial views. (b) Average spinal neural activity induced by the task for each session and group masked by the ROI. *E* and *N*, expert and novices; CB and NB denote sessions; error bars indicate standard error of the mean. * denotes *P* < 0.05.

Additionally, activation in ROI was significantly higher in experts (*P* = 0.024) than in novices when the data from sessions were collapsed (Figure 3(a) and Table 1). This difference in activation was also evident in the comparison between experts and novices in the CB session (*P* = 0.01). We found that across the ROI, the activation increased in expert subjects when they applied controlled breathing, and it decreased in novices under this condition (Figure 3(b)). The interaction of breathing pattern and expertise on IAA-induced neural activity (*E*(CB − NB) > *N*(CB − NB)) was not of significance (*P* = 0.16).

For the secondary mask outside the ROI there was no significant activation for experts (*P* = 0.5), and there was also no significant difference for the contrast of *E*CB − *N*CB (*P* = 0.16).

## 4. Discussion

Our findings supported our hypotheses that the spinal segments innervating an attended-to body part are activated and that expert subjects trigger more neural activity in the corresponding spinal level (L1-2) than do novices. The spinal cord innervates specific body parts, and activation of this area has a rather direct effect on these body parts. Therefore, these results might be an indicator of why or how such practices affect the condition of the body (and not merely the mental condition). Intriguingly, similar activation profile could not be found for the region outside the corresponding spinal segments, meaning that the spinal segments innervating body parts distant from the attended body part are not activated. Previous neuroimaging studies have focused only on the effect of such training on the brain; however, due to the complicated functioning of the brain, the ways in which IAA-related neural activity in the brain affects the body are not simple or straightforward. In other words, whether such changes in the neural activity of the brain influence the body or whether these changes affect only the cognition remains unclear. It is also unclear that, if they influence the body, how they do and in what time frame. Results in this study suggest that there is a direct and instantaneous effect on physical body, but the ability to consciously and consistently accomplish this requires training.

Our results are also interesting from a neuroimaging perspective as this is the first spinal fMRI experiment that did not include motion tasks or sensory stimulation. This means that spinal neurons can be voluntarily activated through mere attention. In fact, our results resemble the demonstration of spinal neuron firing due to motion planning in [34], considering that motion planning could be interpreted as some type of attention towards the body in the service of supporting future movements. The advantage of the spinal fMRI approach used in this study as compared with the methods applied in [34] is that our experiment was totally noninvasive (which was why the previous experiment was performed on monkeys). However, the MRI data alone would not enable straightforward identification of the type of neuronal cells that cause such activation.

Additionally, although for experts the average activation profile was similar across both sessions, it seems that the practice of controlled breathing had a negative effect among novices (but not significant). During the task, some of the novices may not have been able to attend to their body part or may even have experienced tension (compared to rest) due to their lack of experience and mental tension. This could have worsened in the CB session, as additional attention and experience are required to control the breathing pattern. However, expert subjects might be able to volitionally and consistently perform the task in both sessions.

Although the results in this study provide us with some interesting findings, this study has a number of shortcomings and we suggest further studies. First, the short length of the blocks has caused a weak statistical power. The results can only illustrate the activation profile averaged across the ROI, but no individual voxel passed the multiple comparison correction. Second, this study lacks the physiological measurements which might confound the interpretation of changes related to blood flow. On the other hand, since it has been shown that using a constant breathing pattern can reduce the confounds [35] and the same difference of activation between groups was observed during the controlled-breathing session with identical breathing pattern for both groups, we can still expect the results to be valid. Finally, the small number of subjects and also the possibility of contamination of the data with CSF flow, in spite of the percussions taken to avoid it, suggest that the results should not to be taken for granted until they have been replicated across further studies.

## 5. Conclusion

Taken together, results showed greater neural activity in experts. On the other hand, we confirmed this neural activity for only one part of the body; it is important to determine if experts would be able to achieve the same results by attending to other parts of the body. However, we expect that the difference between experts and novices would not be as great for other parts of body, rendering detection difficult.

## Figures and Tables

**Table 1:** Activity patterns within the ROI.

Contrast	*t* value	Size
*E*(CB + NB)	2.11	40
*E*(CB + NB) − *N*(CB + NB)	2.79	181
*E*CB − *N*CB	3.2 (0.08)	249

Cluster peak expressed as the *t* value. “Size” indicates the cluster size in number of voxels. The statistical threshold at the voxel level was set at *P* < 0.05.
